# Data on HO-1 and CD200 protein secretion during T-cells and mesenchymal stromal cells co-cultures

**DOI:** 10.1016/j.dib.2017.02.036

**Published:** 2017-02-21

**Authors:** Mohammad Fayyad-Kazan, Hussein Fayyad-Kazan, Makram Merimi, Nathalie Meuleman, Dominique Bron, Laurence Lagneaux, Mehdi Najar

**Affiliations:** a Institut de Biologie et de Médecine Moléculaires, Université Libre de Bruxelles, 6041 Gosselies, Belgium; b Laboratory of Cancer Biology and Molecular Immunology, Faculty of Sciences I, Lebanese University, Hadath, Lebanon; c Experimental Hematology, Institut Jules Bordet, Université Libre de Bruxelles, 121, Boulevard de Waterloo, 1000, Bruxelles, Belgium; d Laboratory of Clinical Cell Therapy, Institut Jules Bordet, Université Libre de Bruxelles (ULB), Campus Erasme, Brussels, Belgium

**Keywords:** Mesenchymal stromal cells, Activated T-cells, Heme oxygenase-1, CD200

## Abstract

In this *Data in Brief*, we have provided data describing the secretion profile of two main immunoregulatory proteins, heme oxygenase-1 (HO-1) and CD200, from bone marrow (BM), Wharton׳s Jelly (WJ) or adipose tissue (AT) mesenchymal stromal cells (MSCs) being cultivated either in the absence or presence of activated T-cells. Whilst HO-1 is a stress-responsive enzyme displaying diverse cytoprotective effects, CD200 is a membrane glycoprotein delivering immunoregulatory signals following interaction with its receptor (CD200R). Using Enzyme-linked immunosorbent assay (ELISA) techniques, these data are presented to show distinct constitutive secretion of both HO-1 and CD200 depending on MSC types. The data presented also demonstrate that the protein levels of HO-1 and CD200 are differentially modulated during co-culture with activated T-cells. All assays were carried out in triplicates and the mean values are reported. The data presented in this article are complementary to our previously published report entitled “The Immunomodulatory Potential of Mesenchymal Stromal Cells: A Story of a Regulatory Network.” [1].

**Specifications Table**

**Table:** 

Subject area	*Biology*
More specific subject area	Mesenchymal stromal cells (MSCs)
Type of data	Figures
How data was acquired	Enzyme-linked immunosorbent assay (ELISA)
Data format	Analyzed
Experimental factors	HO-1 and CD200
Experimental features	The protein levels of HO-1 and CD200 secretion by MSCs were assessed using ELISA technique
Data source location	Institut Jules Bordet, Brussels, Belgium
Data accessibility	Data are provided in the paper

**Value of the data**

**Table:** 

• The data shown in this article establish the secretion profiles of HO-1 and CD200 in different types of MSCs. • The data analyze the impact of T-cell co-culture on MSC-mediated secretion of HO-1 and CD200. • Constitutive and modulated HO-1 and CD200 secretion levels are differentially observed in these data. • This data helps to better understand the output of the interactions between MSCs and activated T-cells.
• This data will be beneficial for the scientific community focusing on improving the therapeutic capacity of MSCs to modulate the immune response.

## 1 Data

**Fig. 1:**
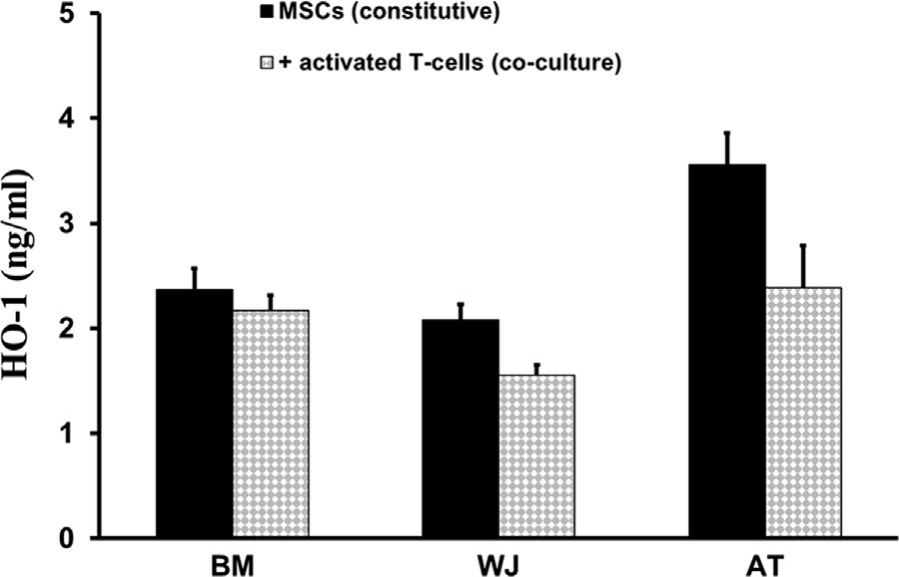
HO-1 production by MSCs. BM-MSCs, WJ-MSCs and AT-MSCs were cultivated in the absence (constitutive) or in the presence of activated T-cells (co-culture). Intracellular HO-1 levels from the cell lysates were then measured using an ELISA kit. Reported values correspond to the mean concentrations (ng/ml)±SD.

**Fig. 2:**
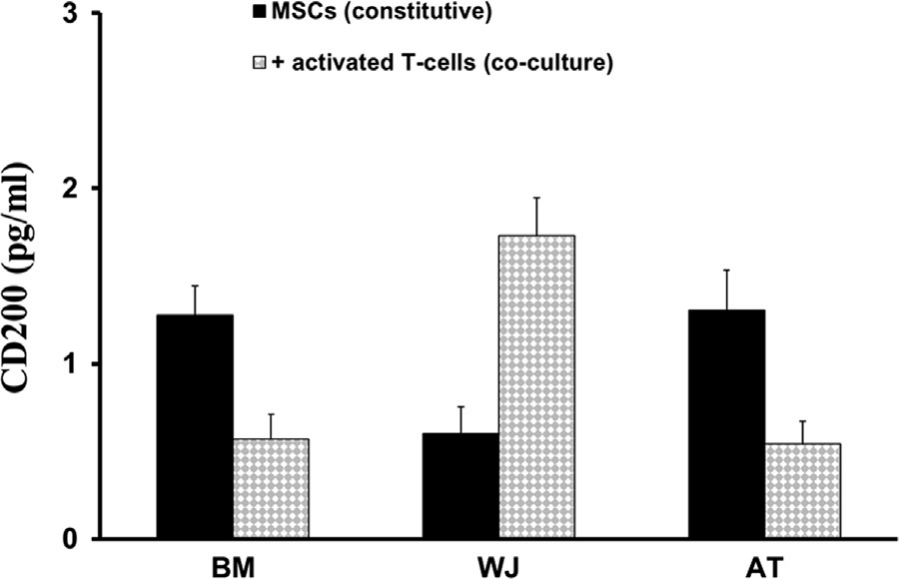
CD200 secretion by MSCs. Following cultivation of BM-MSCs, WJ-MSCs and AT-MSCs in the absence (constitutive) or in the presence of activated T-cells (co-culture), the resulting supernatants were collected and used to assess the levels of CD200 using an ELISA kit. Reported values represent the mean concentrations (pg/ml)±SD.

This data article presents the protein level of HO-1 (Fig. 1) and CD200 (Fig. 2) secreted by BM-MSCs, WJ-MSCs and AT-MSCs in the absence (constitutive) or in the presence of activated T-cells (co-culture).

## 2 Experimental design, materials and methods

### 2.1 Isolation and cultivation of MSCs

This study was conducted in accordance with the Declaration of Helsinki (1964) and approved by the local ethics committee of the “Institut Jules Bordet” (Belgium). The samples were obtained from healthy donors who gave informed written consent. Bone-marrow, adipose tissue and Wharton׳s jelly of the umbilical cord were used to isolate MSCs. Cells were cultured in low glucose Dulbecco׳s Modified Eagle׳s Medium (DMEM-LG, Lonza) supplemented with 15% fetal bovine serum (FBS, Sigma-Aldrich, Bornem, Belgium), 2 mM L-glutamine and 50 U/ml penicillin (both from Lonza) and incubated at 37 °C in a 5% CO_2_ humidified atmosphere to reach 80–90% confluency. Adherent cells were harvested by TrypLE Select (Lonza, Belgium) and expanded by sub culturing at a lower density (1000 cells/cm^2^).

### 2.2 Selection of T-cells

Peripheral blood (PB) cells were obtained from healthy donors after informed consent. Peripheral blood mononuclear cells (PBMCs) were isolated by density gradient centrifugation (LinfoSep, Biomedics, Madrid, Spain). T-cells were purified by positive selection using MACS system (Miltenyi Biotec GmbH, Bergisch, Germany). Selected T-cell purity (CD3^+^), determined by flow cytometry, was always above 95%. Mitogenic stimulation by using 5 µg/ml phytohemagglutinin (PHA, Remel Europe, Kent, UK) with 20 U/ml of interleukin 2 (IL2, Biotest AG, Dreieich, Germany) was carried out to induce T-cell activation.

### 2.3 Co-culture model

Before starting the co-culture, MSCs (25×10^3^) were seeded in flat-bottomed well plates and allowed to adhere overnight. In the next day, activated T-cells (1×10^5^), were added to MSCs for 5 days of co-culture.

### 2.4 Measurement of HO-1 level

After culture, the respective media were removed and the cells were washed twice with PBS (Phosphate Buffered Saline). Adherent MSCs were then harvested by TrypLE Select (Lonza) and centrifuged. The obtained cell pellets were re-suspended in the HO-1 extraction reagent (Enzo Life Sciences, Belgium), being supplemented with a cocktail of protease inhibitors (Roche Applied Science, Belgium), and then incubated for 30 min on ice. The extracts were centrifuged (20,000 g for 10 min at 4 °C) and the supernatants were carefully collected representing thus the cell lysates. HO-1 levels were then quantified using an ELISA kit (Enzo Life Sciences) following the manufacturer׳s instructions.

### 2.5 Measurement of CD200 level

After culture, the respective media were recovered and centrifuged. The resulting supernatants were used to quantify the CD200 level using an ELISA kit (Bio-Connect Diagnostics B.V., Nederland) and following the manufacturer׳s instructions.

### 2.6 Statistical analysis

For each type of MSCs, five different co-cultures were performed. ELISA measurements were carried out in triplicates from and the resulting supernatants. Data are presented as mean±SD (standard deviation) and analysed using Wilcoxon Signed Rank test.
