# Supplementary material for: Data on HO-1 and CD200 protein secretion during T-cells and mesenchymal stromal cells co-cultures
Source: Data Brief. 2017 Feb 21;11:442–5. doi: 10.1016/j.dib.2017.02.036 (PMC5334494; doi:10.1016/j.dib.2017.02.036)
Supplement: Supplementary file 2 — Supplementary material [file mmc2.docx]

|  |  | **HO-1**  **(ng/ml)** | **CD200 (pg/ml)** |
| --- | --- | --- | --- |
| BM-MSCs | Constitutive | 2.4 ± 0.2 | 1.3 ± 0.16 |
|  | + activated T-cells | 2.2 ± 0.15 | 0.6 ± 0.14 |
| WJ-MSCs | Constitutive | 2.1 ± 0.15 | 0.6 ± 0.15 |
|  | + activated T-cells | 1.5 ± 0.1 | 1.7 ± 0.2 |
| AT-MSCs | Constitutive | 3.5 ± 0.3 | 1.3 ± 0.22 |
|  | + activated T-cells | 2.4 ± 0.4 | 0.5 ± 0.13 |
